# Environmental stress increases out-group aggression and intergroup conflict in humans

**DOI:** 10.1098/rstb.2021.0147

**Published:** 2022-05-23

**Authors:** Carsten K. W. De Dreu, Jörg Gross, Lennart Reddmann

**Affiliations:** ^1^ Social, Economic and Organizational Psychology, Leiden University, Leiden, The Netherlands; ^2^ Center for Research in Experimental Economics and Political Decision Making (CREED), University of Amsterdam, Amsterdam, The Netherlands

**Keywords:** carrying-capacity stress, public good provision, parochial altruism, intergroup conflict

## Abstract

Peaceful coexistence and trade among human groups can be fragile and intergroup relations frequently transition to violent exchange and conflict. Here we specify how exogenous changes in groups' environment and ensuing carrying-capacity stress can increase individual participation in intergroup conflict, and out-group aggression in particular. In two intergroup contest experiments, individuals could contribute private resources to out-group aggression (versus in-group defense). Environmental unpredictability, induced by making non-invested resources subject to risk of destruction (versus not), created psychological stress and increased participation in and coordination of out-group attacks. Archival analyses of interstate conflicts showed, likewise, that sovereign states engage in revisionist warfare more when their pre-conflict economic and climatic environment were more volatile and unpredictable. Given that participation in conflict is wasteful, environmental unpredictability not only made groups more often victorious but also less wealthy. Macro-level changes in the natural and economic environment can be a root cause of out-group aggression and turn benign intergroup relations violent.

This article is part of the theme issue ‘Intergroup conflict across taxa’.

## 1. Introduction

Humans operate in groups of familiar others that, in turn, exist next to other groups. Although peaceful coexistence among neighbouring groups is frequently observed [1–3], peaceful intergroup relations are also fragile—it takes only a few individuals in one group to initiate competition with another group and to ignite violent conflict [4–8]. Identifying the reasons for conflict-initiating out-group aggression elucidates the root causes of conflict among human groups and societies and can help to regulate, if not prevent conflict and its waste.

Theory and research on conflict within and between human groups identified a suite of conflict-provoking elements that reside within the intergroup system and are endogenously created and reinforced by past intergroup interactions. There is good evidence, for example, that past conflicts perpetuate in spiteful desire for revenge [6,9], prejudicial misperceptions [10–12] and feelings of in-group superiority and thwarted entitlements [13,14]. Alone and in combination, such psychological states fuel and enable out-group aggression and intergroup conflict [15,16]. As such, past work explains why conflict between human groups easily escalates and why returning to peaceful coexistence can be difficult. What remains poorly understood, however, is why out-group aggression emerges in the first place. Why would groups of people aggress other groups when there is no history of conflict or no disadvantageous inequalities in the distribution of and access to resources?

One possibility, which we examine here, is that out-group aggression can be triggered by factors outside of the intergroup system, like instabilities of the environment and resource availability. If true, changes in environmental predictability and resource availability would explain when and how peaceful coexistence among human groups and societies transitions to violent conflict. Evidence for this possibility would reveal exogenous changes in the environment as a root cause of out-group aggression, and fit observed linkages between macro-level climate change and economic volatilities on the one hand, and the prevalence of political violence, riots and civil wars on the other [17–22]. It would also resonate with work on non-human primate groups engaging in territorial conflicts for food and mating opportunities [23–27].

The starting point in our analysis is that groups operate in a natural, economic and political environment that can be volatile and unpredictable [28,29]. Environmental degradation and resource scarcities alongside erratic and unpredictable fluctuations in living conditions can thus create carrying-capacity stress—situations where the supply of resources is expected to fall short of what groups need or are used to [30–33]. This can have two consequences. First, under environmental threat and unpredictability people form more cohesive groups [34–38] and increase their cooperative contribution to group survival and prosperity [39–41]. Second, and partly because of increased group cohesion and interdependency, group members distance themselves from other groups [36,37,42,43]. Alone and in combination, these processes would lead to in-group bounded, parochial cooperation that serves the in-group and prevents the consumption of resources by members of other groups [9,30,44,45].

Carrying-capacity stress may also motivate parochial competition—tendencies to compete against and harm (members of) out-groups more than (members of) the in-group [9,30,44,46–48]. For example, economic downturn has been associated with increased xenophobic sentiments and inter-ethnic conflict [43,49,50], a rise in nationalistic sentiments [51] and stricter policies for immigration and international trade [49,50,52]. The reason for such increased parochial competition is, first, that environmental threat and unpredictability can make investing in conflict economically more attractive. Resources spent on fighting are typically lost and such ‘investments’ give an uncertain return—even in the case of victory, the spoils of war may only partially off-set the personal contributions made. In unpredictable environments, however, the prospects of appropriating resources from others may compensate for the uncertainty around resource appropriation from the environment. In addition, carrying-capacity stress may lead people to perceive out-groups as comparatively less deserving and entitled [31,53], rendering it justified to contribute to out-group attack and exploitation [54]. Second, carrying-capacity stress may increase vigilance for outside threat, human enemies included [4,42,55,56]. Possibly, carrying-capacity stress leads individuals to contribute to defending the in-group against possible out-group aggression. Accordingly, carrying-capacity stress may lead individuals to contribute private resources to out-group aggression, and perhaps also to in-group defense. It is out-group aggression in particular that ignites and escalates costly intergroup conflict [4,57,58].

We examined whether and how environmental unpredictability and ensuing carrying-capacity stress conditions out-group aggression and in-group defense with experimental contests between attacker and defender groups [4,58,59], and with archival data on militarized disputes between revisionist states aggressing their non-revisionist neighbouring states [59–61]. Across both levels of analysis we find that environmental unpredictability triggers out-group aggression and does not condition in-group defense.

## 2. Intergroup contest experiments

Our experiments implemented intergroup attacker–defender contests (IADC) [4,58,59]. Six individuals were randomly divided into a three-person attacker group and a three-person defender group. Within each group, each individual *i* received 20 Experimental Euros from which they could contribute *g* (0 ≤ *g_i_* ≤ 20) to their group's pool *C* (0 ≤ *C* ≤ 60). Individual contributions to the pool are always wasted. However, when *C*_attacker_ > *C*_defender_, the attacker wins the remaining resources of the defender (60 – *C*_defender_), which is divided equally among attacker group members and added to their remaining endowments (20 – *g*_i_). Defenders thus earn 0 when their attackers win. However, when *C*_attacker_ ≤ *C*_defender_, defenders ‘survive’ and individuals on both sides keep their remaining endowment (20 – *g_i_*). Individual contributions in attacker (defender) groups thus reflect out-group attack (in-group defense) [58].

We created (un)predictable environments by exposing individuals in attacker and/or defender groups to a risk of destruction *u* of their personal endowments (with 0 ≤ *u* ≤ 1), analogous to farmers being uncertain about crop yields, or investors being uncertain about the return on their shares [16,20,28,29,31,32]. If environmental unpredictability increases vigilance, we should see increased conflict expenditures in defender groups when their risk to private property *u* > 0. If environmental unpredictability increases willingness to exploit, we should see increased conflict expenditures in attacker groups when their risk to private property *u* > 0.

The IADC has a unique Nash Equilibrium in mixed strategies [4,58]. Taking the groups as units, with each group being endowed with 20 × 3 = 60 resources, and assuming individuals are risk-neutral rational selfish payoff maximizers, the strategies played in equilibrium imply an average investment in out-group attack (in-group defense) of 5.41 (7.25), and attacker victory in 37.5% of the contest rounds [57,58] (also see §I in the electronic supplementary material). Our experimental manipulation of environmental unpredictability shifts the expected value of contributing to attack and defense upward relative to not contributing. Specifically, when *u* = 0.40, the strategies played in equilibrium imply an average investment in out-group attack (in-group defense) of 10.25 (10.19). Because our manipulation of environmental unpredictability (*u =* 0 versus 0.4) decreases the expected value of keeping resources, it follows that environmental unpredictability should increase out-group attacks. The game-theoretic analyses also show that environmental unpredictability disproportionally increases out-group attack relative to in-group defense, and attacker victory increases to 53.15% in equilibrium. These game-theoretic predictions provide a benchmark against which we can compare investment in out-group aggression and in-group defense in (un)predictable environments. Deviations from these benchmark values would point to additional psychological processes induced by environmental unpredictability, including increased within-group solidarity and feelings of in-group (versus out-group) entitlements.

## 3. Experimental methods and results

Across experiments, we tested 157 male and 305 female subjects in total (mean age ± s.d. = 21.67 ± 1.94). Sample sizes were set *a priori* to fit earlier intergroup contest and public good provision experiments [40,41,58]. For each IADC session, six subjects in individual cubicles were divided at random into a three-person attacker group and a three-person defender group. Since groups were randomly composed of male and female members, gender composition was considered a constant across experimental treatments and not further considered.

Instructions used neutral language throughout (e.g. groups were referred to as Group A and B, contributions were labelled 'investments', and terms like 'in-group defense' and 'out-group attack' were avoided). All subjects passed a comprehension check that consisted of two complete scenarios for one round of the IADC from the perspective of their role, with their group winning and losing the episode, respectively. Thereafter, subjects indicated their contribution *g* (0 ≤ *g_i_* ≤ 20) to their group's pool *C*, and they were informed about the total contribution their group made to *C* (0 ≤ *C* ≤ 60), the total contribution *C* made by the other group, and the total earnings to the members of their own group, themselves included. This concluded one IADC investment round. An IADC session involved 40 (30) contest rounds between an attacker and its defender group in Experiment 1 (Experiment 2).

Experiment 1 had 210 subjects in 35 six-person attacker–defender contests. It compared the baseline IADC [58,59] to a treatment in which the personal endowments of the individuals in the attacker and/or defender group were at risk. In the baseline IADC, everything not invested in attack or defense is for the individual to keep and adds directly to their payment (in the role of defender, only when the group wins the contest). In the unpredictability treatment, individuals in each group faced a risk of destruction *u* of the non-invested individual endowment. Specifically, the endowment not invested was subject to a fixed probability of loss *u* = 0.40. When *u* = 0 (0.40), individuals keep what they do not contribute with probability 1.0 (0.6). The unpredictability parameter *u* was announced before and implemented after each investment round and did not apply to attackers' earnings from the contest.

**Figure 1.**
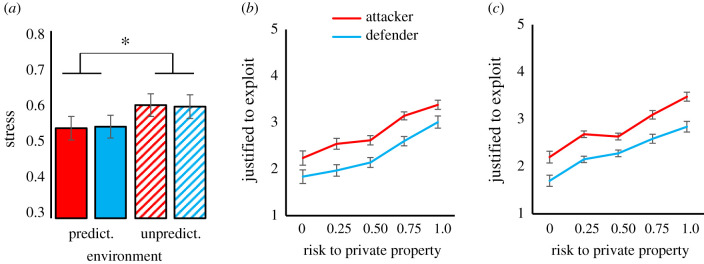
Psychological responses to unpredictable environments. (*a*) Reported stress when the attackers' (red bars) and defenders’ (blue bars) environment is predictable (solid bars) versus unpredictable (dashed bars) (range −2 to +2; displayed *m* ± s.e.); *p ≤ 0.05 (Bonferroni-corrected). **(***b*,*c***)** Justifiability to exploit another group (1 = not at all, to 5 = very much) under different hypothetical probabilities of environmental unpredictability in Experiment 1 and a replication in Experiment 2 (displayed *m* ± s.e.).

We created four blocks of 10 investment rounds each by orthogonally varying *u* (0.00 versus 0.40) for the attacker and defender group, resulting in a 2 (attackers' *u*: 0.40 versus 0.00) × 2 (defenders’ *u*: 0.40 versus 0.00) factorial design. The order in which blocks were presented was controlled in a Latin Square structure and did not influence results. After each 10-round block, subjects indicated how stressed, nervous, tense and worried they felt (all −2 = not at all, to +2 = very much). Ratings were averaged in one index for stress (Cronbach's *α* = 0.76) (our data analytic strategy is described in §2 of the electronic supplementary material). Analysis confirmed that unpredictable environments were experienced as more stressful. Following contest blocks in which their group faced an unpredictable rather than predictable environment, participants reported more stress (figure 1*a*: attackers' environment: *F* = 6.663, *p* = 0.015; defenders’ environment: *F* = 4.702, *p* = 0.037; electronic supplementary material, table S1). In addition, when responding to hypothetical scenarios in which private property was at various levels of risk, individuals felt that out-group exploitation was more justified especially when they were part of attacker groups (figure 1*b,c*; *F* = 16.228, *p* < 0.001).

**Figure 2.**
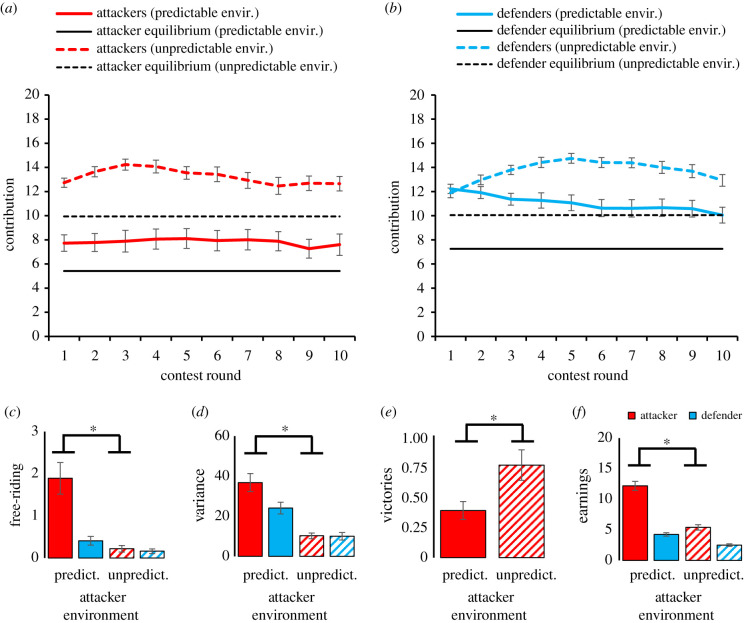
Intergroup conflict when attacker groups are under (un)predictable environments (collapsed across defender group's (un)predictability). (*a*) Contributions to out-group attack (range 0–20; displayed *m* ± s.e.). (*b*) Contributions to in-group defense (range 0–20; displayed *m* ± s.e.). (*c*) Free-riding within groups across contest rounds (range 0–3; displayed *m* ± s.e.), *p ≤ 0.05 (Bonferroni-corrected). (*d*) Within-group variance in contributions as a measure of group coordination; *p ≤ 0.05 (Bonferroni-corrected). (*e*) Victory rates for out-group attacks (proportion out of 10 rounds). (*f*) Post-contest wealth (displayed *m* ± s.e.), *p ≤ 0.05 (Bonferroni-corrected).

In Experiment 1 individuals contributed, on average, less to out-group attack than to in-group defense and, in both cases, above the game-theoretic predictions (figure 2*a,b*). Individuals in defender groups mainly responded to their attackers' behaviour and environmental unpredictability had few effects on in-group defense (electronic supplementary material, tables S2–S4). Furthermore, environmental unpredictability increased out-of-equilibrium investments more in attackers (*m* ± s.e. = 2.415 ± 0.442 to *m* ± s.e. = 3.797 ± 0.741) than in defenders (from *m* ± s.e. = 3.515 ± 0.544 to *m* = 3.678 ± 0.363) (paired *t* = 2.850, *p* = 0.007). Indeed, when attacker groups faced an unpredictable rather than a predictable environment, out-group attack increased substantially from *m* ± s.e. = 7.825 ± 0.741 to *m* ± s.e. = 11.035 ± 0.544 (attacker's environment × role: *F* = 40.762, *p* < 0.001). This gives some first indication that carrying-capacity stress increases out-group attack in particular, and beyond what a game-theoretic analysis based on a shift of expected value of conflict would predict. Possibly, therefore, out-group attack is also motivated by additional psychological processes such as a heightened sense of in-group solidarity and considerations of entitlement.

Environmental unpredictability already influenced out-group attack early in the contest (figure 2*a*,*b*; attacker's environment × role × round, *F* = 3.592, *p* = 0.005) and independently of the defender's environmental (un)predictability (electronic supplementary material, tables S2–S4). On the first contest round, when groups were unaware of how others would behave and environmental unpredictability had not been effectuated, out-group attack was already stronger under environmental unpredictability (*F* = 48.841, *p* < 0.001; role × attacker environment, *F* = 41.417, *p* < 0.001). Defense was not significantly influenced by environmental unpredictability (*F* = 41.417, *p* = 0.652; role × defender environment, *F* = 2.4179, *p* = 0.125).

Analyses of within-group dynamics and individual decisions revealed further support for the possibility that environmental unpredictability increases parochialism especially during out-group attack. Individuals in attacker groups free-rode less frequently (i.e. investing 0 in the conflict) when their environment was unpredictable (figure 2*c*: attacker's environment × role, *F* = 22.127, *p* < 0.001). Individuals in attacker groups facing unpredictable environments also reduced within-group variance in contributions, suggesting improved coordination of collective action (figure 2*d*; attacker's environment × role, *F* = 7.959, *p* = 0.008; electronic supplementary material, tables S3–S4). Hence, environmental unpredictability intensified intergroup conflict because it increased conflict participation and within-group coordination in attacker groups. Within-group coordination is difficult to explain from the perspective of expected value analysis, and results give further support to the possibility that carrying-capacity stress can lead to conflict (also) because it increases group cohesion and within-group solidarity.

Under unpredictable environments, out-group attacks were successful almost twice as frequently (from 39.2% to 75.8%; *F* = 20.734, *p* < 0.0001; figure 2*e*). Whereas attacker victory rate fits the game-theoretic equilibrium of 37.5% for predictable environments (one-sample *t* = 0.148, *p* = 0.883), it significantly exceeds the predicted 53.15% in unpredictable environments (one-sample *t* = 1.988, *p* = 0.048). At the same time, attacker groups exposed to unpredictable environments earned significantly less than those operating under predictable conditions (figure 2*f*; attackers' environment × role; *F* = 144.789, *p* < 0.001; electronic supplementary material, tables S3–S4). Thus, while groups react to unpredictability by intensifying their attempts to take advantage of another group, they end up less wealthy. Furthermore, environmental unpredictability reduces relative wealth differences between attacker and defender groups—environmental unpredictability makes groups not only aggressive and victorious, but also poor.

Results for earnings and victory rates suggest that out-group aggression triggered by unpredictable environments is maladaptive from a (within-group) welfare perspective yet adaptive from a relative gain perspective—it makes groups more frequently victorious. Perhaps unpredictable environments and ensuing carrying-capacity stress shift the group members’ goal from maximizing wealth to maximizing winning. This resonates with studies showing that stress due to cognitive load and time pressure lead to more aggressive attacks and lower earnings, yet higher victory-rates [54]. Furthermore, such a ‘glow of winning’ is associated with activation in brain regions related to value computation (i.e. ventral striatum) [62] and reduced activation in brain regions typically associated with controlled cost–benefit analysis and impulse inhibition (i.e. dorsolateral prefrontal cortex) [59].

A possible concern with Experiment 1 is that all members in one group faced the same level of unpredictability. This shared common fate may in itself create and increase group cohesion [35–38]. Accordingly, it may be shared common fate rather than unpredictable environments that led to increased parochialism and out-group aggression. We examined this possibility in Experiment 2, with 252 subjects in 42 contests. Methods and materials were identical to those in Experiment 1, with a few exceptions. Defender groups were always under *u* = 0 and attacker groups were always under *u* = 0.40 on average. Second, environmental unpredictability *u* was conditional on contest success: if an attacker group won (lost) a contest round, each member's *u* decreased (increased) with 0.05 (until *u* plateaued at 0 or 1). However, because victory rate (49.3%) did not change across the contest (*F* = 1.148, *p* = 0.417), attacker groups mostly maintained a stationary environmental unpredictability at *u* ≈ 0.40 and this element in the experiment is subsequently ignored (electronic supplementary material, table S5).

We decomposed a shared common fate and an environmental unpredictability explanation by manipulating, within attacker groups, the individual's personal risk of destruction. In 21 attacker groups, environmental unpredictability was the same for each group member (*u* = 0.40). In another 21 attacker groups, unpredictability differed across group members: One member of an attacker group was under *u* = 0.20, one was exposed to *u* = 0.40, and the third group member was exposed to *u* = 0.60. If shared common-fate rather than environmental unpredictability explains the results of Experiment 1, we should see stronger parochialism in attacker groups with aligned (i.e. *u* = [0.4, 0.4, 0.4]) compared to misaligned (i.e. *u* = [0.2, 0.4, 0.6]) risk to private property.

**Figure 3.**
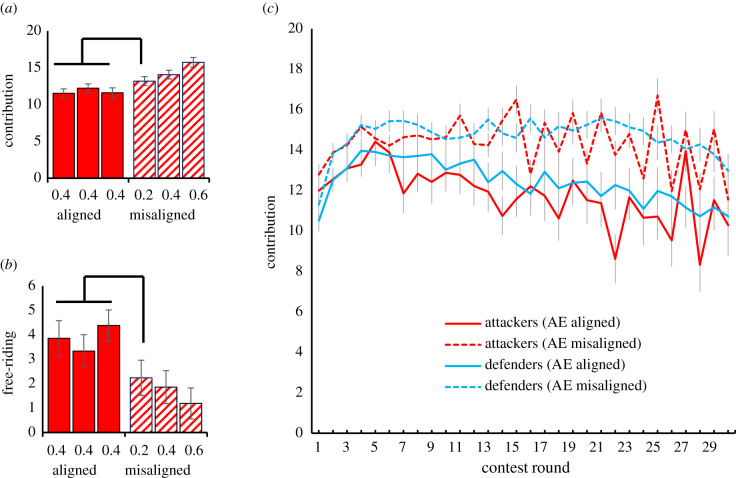
Group dynamics in attacker groups when they face aligned versus misaligned environmental unpredictability (AE), with all members in the aligned condition being at round 1 under *u* = 0.4, and in the misaligned conditions being at *u* = 0.2 versus 0.4 versus 0.6. (*a*) Average individual contributions (range 0–20; displayed *m* ± s.e.). (*b*) Free-riding per individual across contest rounds (range 0–30; displayed *m* ± s.e.); bars linked with connectors are significantly different at Bonferroni-corrected *p* ≤ 0.05. (*c*) Contributions to out-group attack and in-group defense over time (range 0–20; displayed *m* ± s.e.).

Results do not support the common fate explanation: out-group attack was higher on average when environmental unpredictability was misaligned rather than aligned ([mis]alignment *F* = 12.429, *p* = 0.001; member × [mis]alignment: *F* = 5.462, *p* = 0.006; figure 3*a*) (electronic supplementary material, tables S7 and S8). Crucially, individuals in the misaligned condition with lower unpredictability (i.e. *u* = 0.20) contributed more to out-group attack than individuals in the aligned condition (facing higher unpredictability, i.e. *u* = 0.40; figure 3*a*). The same pattern was observed for free-riding, which was lower under misaligned environmental unpredictability (*F* = 6.308, *p* = 0.016) and lower among individuals with low unpredictability in the misaligned condition (*u* = 0.20) than those with higher unpredictability (*u* = 0.40) in the aligned condition (figure 3*b*: member × [mis]alignment: *F* = 2.996, *p* = 0.091; marginal). Hence, individuals in the misaligned condition facing lower risks ‘stood by’ their group members facing higher risks. Such a display of within-group solidarity is difficult to explain from an expected value perspective. It fits, however, the hypothesis that unpredictable environments increase parochialism and intergroup conflict, even for those individuals that are less affected [8,63].

At the aggregate level, we replicated Experiment 1. Contributions were lower in attacker compared to defender groups (*F* = 12.071, *p* = 0.001), and both attack and defense were out-of-equilibrium. Furthermore, we found that contributions to out-group attack were higher when environmental unpredictability was misaligned (*F* = 11.856, *p* = 0.001; figure 3*c*). Under aligned unpredictability, attack intensified early on and then steadily declined. When unpredictability was misaligned, however, contributions to attack increased and then stabilized (round × [mis]alignment: *F* = 2.720, *p* = 0.035; electronic supplementary material, tables S7 and S8).

## 4. Archival analysis of interstate conflict

**Figure 4.**
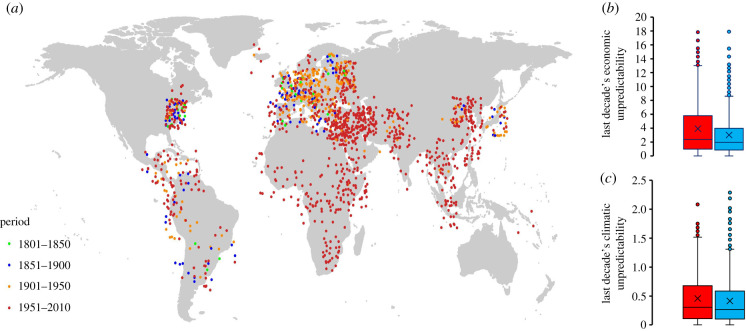
World map with 1,474 interstate disputes showing revisionist aggressors (dots, jittered per country) and their non-revisionist defenders (green = 1800–1851; blue = 1851–1900; orange = 1901–1950; red = 1951–2010). In the decade prior to conflict onset, revisionist aggressors (red bars) faced greater fluctuations in *per capita* economic productivity (*b*) and in annual surface temperature (*c*) than their non-revisionist ‘defenders’ (blue bars). (Box-plots based on (*b*) 1427 and (*c*) 1053 conflict pairs, respectively, with whiskers showing standard deviation around the mean [×] and median (horizontal bars within the boxes).)

At the outset, we noted that archival and macroeconomic data documented that environmental shocks link to nationalistic sentiments [43,49–52,64] and that nationalism links to hawkish foreign policy [10,51,64]. Our experiments reveal the possible micro-foundations of such macrolevel tendencies by showing how environmental unpredictability and concomitant carrying-capacity stress strengthens within-group solidarity and the ability to align behaviour in well-coordinated collective action. Possibly, carrying-capacity stress also promotes the formation of and compliance with in-group norms and leader initiatives aimed at aggressing neighbouring communities and nation states [38,65]. Local sentiments and parochial interests among groups and communities within an overarching nation state may support and fuel nationalism and support for hawkish foreign policy [10,31,60,64–67]. If true, we should see that environmental unpredictability and ensuing carrying-capacity stress associates with enhanced probability of nations aggressing their neighbours more than with nations defending against their neighbour's aggression. We examined this possibility in archival analyses of 1447 militarized disputes [58,60,61] between revisionist ‘aggressor’ states and their non-revisionist ‘defenders’ (figure 4*a*; electronic supplementary material §III).

We first looked at environmental unpredictability in terms of economic productivity. For each country within 1053 aggressor−defender pairs we created *per capita* economic productivity growth vectors for the ten years prior to the onset of the dispute [68,69] (electronic supplementary material §III). Each value within each country's 10-year series was expressed as the percentage deviation from the 10-year mean. We fitted regression lines to these adjusted values and used the residual standard deviation to index economic variability. Fitting the micro-level dynamics uncovered in our experiments, we find that in the decade prior to conflict onset, aggressors compared to their defenders experienced higher economic variability (*b* ± se = 0.6547 ± 0.1543, *p* < 0.001; figure 4*b*). Next we considered environmental unpredictability in terms of climatic variability. For 1427 aggressor−defender pairs, we obtained the hemispheric coordinates of each country's capital city at the onset of the conflict [70] and matching 10-year annual surface temperature vectors [71] (electronic supplementary material §II). We fitted linear regression models to each 10-year temperature vector within each country and took the residual standard deviation as a measure of climatic variability. We find that in the decade prior to conflict onset, aggressors compared to their defenders experienced more climatic variability (*b* ± se = 0.093 ± 0.016, *p* < 0.001; figure 4*c*; electronic supplementary material, tables S9–S11).

## 5. Conclusion

The results of our experiments taken together show that when risk to private property is shared with other group members, individuals feel stressed and justified to extract resources from another group. When environmental unpredictability increased, individuals contributed more to their group's efforts to exploit other groups through coordinated out-group attacks and were more victorious. These effects of unpredictable environments on out-group attacks emerged already in the first round of the intergroup contest (figures 1*a* and 4*c*; electronic supplementary material, tables S3 and S7A). At this point in the contest, the strength of defense is unknown and environmental unpredictability has not been effectuated. This suggests that groups respond to unpredictable environments preemptively by initiating conflict with another group.

To some extent, group members may be more motivated to contribute resources to intergroup conflict when the environment is unpredictable as the expected value of private property is reduced. This possibility fits game-theoretic predictions. Importantly, however, we observed significant over-investment compared to risk-neutral rational selfish play, especially for out-group attack. That contributions and attacker victory rates were persistently out-of-equilibrium reveals that rational selfish play cannot fully explain results when the environment is either predictable or unpredictable. Instead, results fit earlier work suggesting that the stress induced by environmental unpredictability focuses investments towards group winning rather than individual or group-level profit-maximization [54,57,59]. Results showed that at the group level, environmental unpredictability strengthens within-group solidarity and effective coordination of collective action aimed at exploiting rivaling out-groups.

Groups respond to unpredictable environments by fighting other groups, both in laboratory experiments and at the interstate level. While the ‘spoils of war’ from victories and the ‘glow of winning’ may provide some immediate reward, our results suggest that unpredictable environments lead to out-group attack and in-group defense that substantially reduce individual welfare and collective efficiency. In our experiments, groups facing predictable environments wasted 53% of their collective resource on intergroup conflict. When the environment became unpredictable, waste increased to 82% (and 75% in Experiment 2).

Our experimental set-up allowed individual group members to contribute either to out-group attacks or to in-group defense. There are situations in which individuals can do both to varying degrees, or in an alternating fashion between defending and attacking. Even within groups, some may contribute resources to in-group defense while others contribute to out-group attack. Our experiments teased apart out-group attack and in-group defense to cleanly estimate the possible impact of environmental unpredictability and concomitant carrying-capacity stress. It allows us to conclude that changes in the group's environment condition out-group aggression more than in-group defense. Future research could examine this conclusion when individuals are able to contribute to both defense and attack. In addition, our experimental set-up allowed groups no other means to serve group welfare than to attack out-groups. Peaceful solutions to unpredictable environments would mean that groups refrain from investing in out-group attack and collectively ‘free-ride’ during the contest. Alternative solutions, including investing in local group goods and innovation, are sometimes available as well. Future studies could examine environments that allow groups under varying levels of environmental unpredictability to choose between peaceful and aggressive options to serve their group. There is some evidence that group innovation in science and technology increases under harsher, more unpredictable environments [72] but whether and to what extent possibilities for group innovation reduce out-group attack and intergroup conflict as a response to unpredictable environments remains an open question.

Conflict theory and research in the social and behavioural sciences explain the prevalence and re-occurrence of conflict between human groups and societies mainly in terms of elements inherent to and endogenously created by histories of competitive intergroup exchanges [4–16]. Consistent with recent work in political geography and climate science [17–22], and on intergroup conflict among non-human animals [23–25], we identified here when and how ecological risks, and changes therein, can lead humans to contribute to out-group aggression. While pro-social inclinations to reduce the in-group's carrying-capacity stress may motivate such out-group aggression, it turns potentially benign intergroup relations into inherently wasteful conflict.

## Data Availability

All data and computing scripts are deposited in Dataverse https://doi.org/10.34894/HZD5NV. Full analytic models and results are provided in electronic supplementary material [73].

## References

[1] Fry DP, Soderberg P. 2013 Lethal aggression in mobile forager bands and implications for the origins of war. Science **341**, 270-273. (10.1126/science.1235675) 23869015

[2] Handley C, Mathew S. 2020 Human large-scale cooperation as a product of competition between cultural groups. Nat. Commun. **11**, 702. (10.1038/s41467-020-14416-8) 32019930 PMC7000669

[3] Gross J, De Dreu CKW. 2019 The rise and fall of cooperation through reputation and group polarization. Nat. Commun. **10**, e776. (10.1038/s41467-019-08727-8) PMC6377668 30770812

[4] De Dreu CKW, Gross J. 2019 Revisiting the form and function of conflict: neurobiological, psychological and cultural mechanisms for attack and defense within and between groups. Behav. Brain Sci. **42**, 1-42. (10.1017/S0140525X19000037) 30251617

[5] De Dreu CKW. 2010 Social conflict: the emergence and consequences of struggle and negotiation. In Handbook of social psychology (eds ST Fiske, DT Gilbert, G Lindzey), pp. 983-1023. New York, NY: Wiley. (10.1002/9780470561119.socpsy002027)

[6] Lickel B, Miller N, Stenstrom DM, Denson TF, Schmader T. 2006 Vicarious retribution: the role of collective blame in intergroup aggression. Pers. Soc. Psychol. Rev. **10**, 372-390. (10.1207/s15327957pspr1004_6) 17201594

[7] Glowacki L, Isakov A, Wrangham RW, McDermott R, Fowler JH, Christakis NA. 2016 Formation of raiding parties for intergroup violence is mediated by social network structure. Proc. Natl Acad. Sci. USA **113**, 12 114-12 119. (10.1073/pnas.1610961113) 27790996 PMC5086992

[8] Doğan G, Glowacki L, Rusch H. 2018 Spoils division rules shape aggression between natural groups. Nat. Hum. Behav. **2**, 322-326. (10.1038/s41562-018-0338-z) 30962600

[9] Brewer MB. 1999 The psychology of prejudice: in-group love and out-group hate? J. Soc. Issues **55**, 429-444. (10.1111/0022-4537.00126)

[10] Gat A. 2013 Nations: The long history and deep roots of political ethnicity and nationalism. Cambridge, UK: Cambridge University Press.

[11] Whitehouse H, McQuinn B, Buhrmester M, Swann WB. 2014 Brothers in arms: Libyan revolutionaries bond like family. Proc. Natl Acad. Sci. USA **111**, 17 783-17 785. (10.1073/pnas.1416284111) PMC4273349 25385591

[12] Bar-Tal D. 2007 Sociopsychological foundations of intractable conflicts. Am. Behav. Sci. **50**, 1430-1453. (10.1177/0002764207302462)

[13] Porat R, Halperin E, Tamir M. 2016 What we want is what we get: group-based emotional preferences and conflict resolution. J. Pers. Soc. Psychol. **110**, 167-190. (10.1037/pspa0000043) 26785061

[14] Ginges J. 2019 The moral logic of political violence. Trends Cogn. Sci. **23**, 1-3. (10.1016/j.tics.2018.11.001) 30497936

[15] Alarcon R, Yost J, Erickson P, Beckman S. 2019 The proximate causes of Waorani warfare. Hum. Nat. **30**, 247-271. (10.1007/s12110-019-09348-2) 31313088

[16] Grant PR, Brown R. 1995 From ethnocentrism to collective protest: responses to relative deprivation and threats to social identity. Soc. Psychol. Quart. **58**, 195-212. (10.2307/2787042)

[17] Kennett DJ, Marwan N. 2015 Climatic volatility, agricultural uncertainty, and the breakdown of preindustrial agrarian states. Phil. Trans. R. Soc. A **373**, 20140458. (10.1098/rsta.2014.0458) 26460110

[18] Lee HF. 2018 Internal wars in history: triggered by natural disasters or socio-ecological catastrophes? Holocene **28**, 1071-1081. (10.1177/0959683618761549)

[19] O'Loughlin J, Linke AM, Wimer FDM. 2014 Effects of temperature and precipitation variability on the risk of violence in sub-Saharan Africa, 1980–2012. Proc. Natl Acad. Sci. USA **111**, 16 712-16 717. (10.1073/pnas.1411899111) PMC4250158 25385621

[20] Von Uexkull N, Croicu M, Fjelde H, Buhaug H. 2016 Civil conflict sensitivity to growing-season drought. Proc. Natl Acad. Sci. USA **113**, 12 391-12 396. (10.1073/pnas.1607542113) 27791091 PMC5098672

[21] Hsiang SM, Burke M, Miguel E. 2013 Quantifying the influence of climate on human conflict. Science **341**, 1212. (10.1126/science.1235367) 24031020

[22] Schleussner C-F, Donges JF, Donner RV, Schellnhuber HJ. 2016 Armed-conflict risks enhanced by climate-related disasters in ethnically fractionalized countries. Proc. Natl Acad. Sci. USA **113**, 9216-9221. (10.1073/pnas.1601611113) 27457927 PMC4995947

[23] Golabek KA, Ridley AR, Radford AN. 2012 Food availability affects strength of seasonal territorial behavior in a cooperatively breeding bird. Anim. Behav. **83**, 613-619. (10.1016/j.anbehav.2011.11.034)

[24] Scarry CJ. 2017 Male resource defence during intergroup aggression among tufted capuchin monkeys. Anim. Behav. **123**, 169-178. (10.1016/j.anbehav.2016.10.015)

[25] Lemoine S, Preis A, Samuni L, Boesch C, Crockford C, Wittig RM. 2020 Between-group competition impacts reproductive success in wild chimpanzees. Curr. Biol. **30**, 312. (10.1016/j.cub.2019.11.039) 31902731 PMC6971690

[26] Brown M, Steinitz R, Emery Thompson M. 2022 Wins and losses in intergroup conflicts reflect energy balance in red-tailed monkeys. Phil. Trans. R. Soc. B **377**, 20210152. (10.1098/rstb.2021.0152) 35369757 PMC8977655

[27] García MG, de Guinea M, Bshary R, van de Waal E. 2022 Drivers and outcomes of between-group conflict in vervet monkeys. Phil. Trans. R. Soc. B **377**, 20210145. (10.1098/rstb.2021.0145) 35369750 PMC8977665

[28] Frankenhuis WE, Pranchanathan K, Nettle D. 2016 Cognition in harsh and unpredictable environments. Curr. Opin. Psych. **7**, 76-80. (10.1016/j.copsyc.2015.08.011)

[29] Duncan RB. 1972 Characteristics of organizational environments and perceived environmental uncertainty. Adm. Sci. Q. **17**, 313-327. (10.2307/2392145)

[30] De Dreu CKW, Gross J, Farina A, Ma Y. 2020 Group cooperation, carrying-capacity stress, and intergroup conflict. Trends Cogn. Sci. **24**, 760-776. (10.1016/j.tics.2020.06.005) 32620334

[31] Ember CR, Ember M. 1992 Resource unpredictability, mistrust, and war: a cross-cultural study. J. Confl. Res. **36**, 242-262. (10.1177/0022002792036002002)

[32] Ellis BJ, Figueredo AJ, Brumbauch BH, Schlomer GL. 2009 Fundamental dimensions of environmental risk. Hum. Nat. **20**, 204-268. (10.1007/s12110-009-9063-7) 25526958

[33] Read DW, LeBlanc SA. 2003 Population growth, carrying capacity, and conflict. Curr. Anthrop. **44**, 59-85. (10.1086/344616)

[34] Landau MJ, Kay AC, Whitson JA. 2015 Compensatory control and the appeal of a structured world. Psych. Bull. **141**, 594. (10.1037/a0038703) 25688696

[35] Spencer-Rogers J, Hamilton DL, Sherman SJ. 2007 The central role of entitativity in stereotypes of social categories and task groups. J. Pers. Soc. Psych. **92**, 369-388. (10.1037/0022-3514.92.3.369) 17352598

[36] Kruglanski AW, Pierro A, Mannetti L, De Grada E. 2006 Groups as epistemic providers: need for closure and the unfolding of group-centrism. Psych. Rev. **113**, 84-100. (10.1037/0033-295X.113.1.84) 16478302

[37] Hogg MA. 2007 Uncertainty–identity theory. Adv. Exp. Soc. Psychol. **39**, 69-126. (10.1016/S0065-2601(06)39002-8)

[38] Barth M, Masson T, Fristsche I, Ziemer CT. 2018 Closing ranks: Ingroup norm conformity as a subtle response to threatening climate change. Group Process. Intergr. Relat. **21**, 497-512. (10.1177/1368430217733119)

[39] Calo-Blanco A, Kovářík J, Mengel F, Romero JG. 2017 Natural disasters and indicators of social cohesion. PloS ONE **12**, e0176885-13. (10.1371/journal.pone.0176885) 28591148 PMC5462365

[40] Gross J, De Dreu CKW. 2019 Individual solutions to shared problems create a modern tragedy of the commons. Sci. Adv. **5**, eaau7296. (10.1126/sciadv.aau7296) 31001579 PMC6469947

[41] Gross J, Veistola S, De Dreu CKW, Van Dijk E. 2020 Self‐reliance crowds out group cooperation and increases wealth inequality. Nat. Commun. **11**, 1561. (10.1038/s41467-020-18896-6) 33057001 PMC7560835

[42] De Dreu CKW, Greer LL, Handgraaf MJJ, Shalvi S, Van Kleef GA, Baas M, Ten Velden FS, Van Dijk E, Feith SWW. 2010 The neuropeptide oxytocin regulates parochial altruism in intergroup conflict among humans. Science **328**, 1408-1411. (10.1126/science.1189047) 20538951

[43] Krosch AR, Tyler TR, Amodio DM. 2017 Race and depression: effects of economic scarcity on racial discrimination. J. Pers. Soc. Psych. **113**, 892-909. (10.1037/pspi0000112) 28910122

[44] Balliet D, Wu J, De Dreu CKW. 2014 In-group favoritism in cooperation: a meta-analysis. Psych. Bull. **140**, 1556-1581. (10.1037/a0037737) 25222635

[45] Yamagishi T, Mifune N. 2016 Parochial altruism: does it explain modern human group psychology? Curr. Opin. Psychol. **7**, 39-43. (10.1016/j.copsyc.2015.07.015)

[46] Choi J-K, Bowles S. 2007 The coevolution of parochial altruism and war. Science **318**, 636-640. (10.1126/science.1144237) 17962562

[47] Bornstein G. 2003 Intergroup conflict: individual, group, and collective interests. Pers. Soc. Psych. Rev. **7**, 129-145. (10.1207/S15327957PSPR0702_129-145) 12676644

[48] Halevy N, Bornstein G, Sagiv L. 2008 ‘In-Group Love’ and ‘Out-Group Hate’ as motives for individual participation in intergroup conflict: a new game paradigm. Psychol. Sci. **19**, 405-411. (10.1111/j.1467-9280.2008.02100.x) 18399895

[49] Van der Ploeg F, Poelhekke S. 2009 Volatility and the natural resource curse. Oxf. Econ. Papers **61**, 727-760. (10.1093/oep/gpp027)

[50] Colantone I, Stanig P. 2018 The trade origins of economic nationalism: import competition and voting behavior in Western Europe. Am. J. Political Sci. **62**, 936-953. (10.1111/ajps.12358)

[51] Bertoli AD. 2015 Nationalism and conflict: lessons from international sports. Int. Stud. Q. **61**, 835-849. (10.1093/isq/sqx029)

[52] Margalit Y. 2019 Political responses to economic shocks. Annu. Rev. Political Sci. **22**, 277-295. (10.1146/annurev-polisci-050517-110713)

[53] Rai TS, Valdesolo P, Graham J. 2017 Dehumanization increases instrumental violence, but not moral violence. Proc. Natl Acad. Sci. USA **114**, 8511-8516. (10.1073/pnas.1705238114) 28739935 PMC5559031

[54] De Dreu CKW, Giacomantonio M, Giffin MR, Vechiatto G. 2019 Psychological constraints on aggressive predation in economic contests. J. Exp. Psych.: G **148**, 1767-1781. (10.1037/xge0000531) 30556723

[55] Jonas E, McGregor I, Klackl J, Agroskin D, Fritsche I, Holbrook C, Nash K, Proulx T, Quirin M. 2014 Threat and defense: from anxiety to approach. Adv. Exp. Soc. Psychol. **49**, 219-286. (10.1016/B978-0-12-800052-6.00004-4)

[56] Böhm R, Rusch H, Güreck O. 2016 What makes people go to war? Defensive intentions motivate retaliatory and preemptive intergroup aggression. Evol. Hum. Behav. **37**, 29-34. (10.1016/j.evolhumbehav.2015.06.005)

[57] De Dreu CKW, Pliskin R, Rojek-Giffin M, Meder Z, Gross J. 2021 Political games of attack and defense. Phil. Trans. R. Soc **376**, 20200135. (10.10968/rstb.2020.0135) PMC7934902 33611990

[58] De Dreu CKW, Gross J, Meder Z, Griffin MR, Prochazkova E, Krikeb J, Columbus S. 2016 In-group defense, out-group aggression, and coordination failure in intergroup conflict. Proc. Natl Acad. Sci. USA **113**, 10 524-10 529. (10.1073/pnas.1605115113) 27601640 PMC5035908

[59] Yang J, Zhang H, Ni J, De Dreu CKW, Ma Y. 2020 Within-group neural synchronization in the prefrontal cortex associates with intergroup conflict. Nat. Neurosci. **23**, 754-760. (10.1038/s41593-020-0630-x) 32341541

[60] Wright TM. 2014 Territorial revision and state repression. J. Peace Res. **51**, 375-387. (10.1177/0022343314520822)

[61] Jones DM, Bremer SA, Singer JD. 1996 Militarized interstate disputes 1816–1992: rationale, coding rules, and empirical patterns. Confl. Manag. Peace Sci. **15**, 163-215. (10.1177/073889429601500203)

[62] Rojek-Giffin MR, Lebreton M, Scholte HS, Van Winden F, Ridderinkhof R, De Dreu CKW. 2020 Neurocognitive underpinnings of aggressive predation in economic contests. J. Cogn. Neurosci. **32**, 1276-1288. (online first). (10.1162/jocn_a_01545) 32073348

[63] Theelen MMP, Bohm R. 2020 The conflict-cooperation effect persists under intragroup payoff asymmetry. Group Process. Intergr. Relat. **24**, 815-835. (early access). (10.1177/1368430220910795)

[64] Cederman LE, Warren TC, Sornette D. 2011 Testing Clausewitz: nationalism, mass mobilization, and the severity of war. Int. Organ. **65**, 605-638. (10.1017/S0020818311000245)

[65] Waldman DA, Ramirez GG, House RJ, Puranam P. 2001 Does leadership matter? CEO leadership attributes and profitability under conditions of perceived environmental uncertainty. Acad. Manag. J. **44**, 134-143. (10.5465/3069341)

[66] Baker WD, Oneal JR. 2001 Patriotism or opinion leadership? The nature and origins of the "rally ‘round the flag" effect. J. Confl. Resol. **45**, 661-687. (10.1177/0022002701045005006)

[67] Bonikowski B. 2016 Nationalism in settled times. Annu. Rev. Sociol. **42**, 427-449. (10.1146/annurev-soc-081715-074412)

[68] Bolt J, Inklaar R, de Jong H, van Zanden JL. 2018 Rebasing ‘Maddison’: new income comparisons and the shape of long-run economic development. Maddison Project Working paper, **10**. (10.1177/0022002701045005006)

[69] Feenstra RC, Inklaar R, Timmer MP. 2015 The Next Generation of the Penn World Table. Am. Econ. Rev. **105**, 3150-3182. (10.1257/aer.20130954)

[70] Hensel P. 2018 ICOW Historical State Names Data Set. Retrieved on June 2018 from http://www.paulhensel.org/icownames.html.

[71] Rohde R, Muller R, Jacobsen R, Perlmutter S, Mosher S. 2013 Berkeley Earth Temperature averaging process. Geoinformatics Geostat.: Overv. **1**, 1-3. (10.4172/2327-4581.1000103)

[72] De Dreu CKW, Van Dijk MA. 2018 Climatic shocks associate with innovation in science and technology. PLoS ONE **13**, e0190122. (10.1371/journal.pone.0190122) 29364910 PMC5783359

[73] De Dreu CKW, Gross J, Reddmann L. 2022 Environmental stress increases out-group aggression and intergroup conflict in humans. *Figshare*. 10.1098/rstb.2021.0147 PMC8977653 35369744

